# Engineering In-Co_3_O_4_/H-SSZ-39(OA) Catalyst for CH_4_-SCR of NO_x_: Mild Oxalic Acid (OA) Leaching and Co_3_O_4_ Modification

**DOI:** 10.3390/molecules29163747

**Published:** 2024-08-07

**Authors:** Guanyu Chen, Weixin Zhang, Rongshu Zhu, Yanpeng Chen, Minghu Zhao, Mei Hong

**Affiliations:** 1 State Key Laboratory of Urban Water Resource and Environment, Shenzhen Key Laboratory of Organic Pollution and Control, School of Civil and Environmental Engineering, Harbin Institute of Technology, Shenzhen 518055, China; 2 Guangdong Provincial Key Laboratory of Nano-Micro Materials Research, School of Advanced Materials, Peking University Shenzhen Graduate School (PKUSZ), Shenzhen 518055, China

**Keywords:** SSZ-39, CH_4_-SCR, acid leaching, post-treatment, Co_3_O_4_, zeolite-based catalyst

## Abstract

Zeolite-based catalysts efficiently catalyze the selective catalytic reduction of NO*_x_* with methane (CH_4_-SCR) for the environmentally friendly removal of nitrogen oxides, but suffer severe deactivation in high-temperature SO_2_- and H_2_O-containing flue gas. In this work, SSZ-39 zeolite (AEI topology) with high hydrothermal stability is reported for preparing CH_4_-SCR catalysts. Mild acid leaching with oxalic acid (OA) not only modulates the Si/Al ratio of commercial SSZ-39 to a suitable value, but also removes some extra-framework Al atoms, introducing a small number of mesopores into the zeolite that alleviate diffusion limitation. Additional Co_3_O_4_ modification during indium exchange further enhances the catalytic activity of the resulting In-Co_3_O_4_/H-SSZ-39(OA). The optimized sample exhibits remarkable performance in CH_4_-SCR under a gas hourly space velocity (GHSV) of 24,000 h^−1^ and in the presence of 5 vol% H_2_O. Even under harsh SO_2_- and H_2_O-containing high-temperature conditions, it shows satisfactory stability. Catalysts containing Co_3_O_4_ components demonstrate much higher CH_4_ conversion. The strong mutual interaction between Co_3_O_4_ and Brønsted acid sites, confirmed by the temperature-programmed desorption of NO (NO-TPD), enables more stable N*_x_*O*_y_* species to be retained in In-Co_3_O_4_/H-SSZ-39(OA) to supply further reactions at high temperatures.

## 1. Introduction

The selective catalytic reduction of NO*_x_* with CH_4_ (CH_4_-SCR) has attracted considerable interest because it is capable of simultaneously abating harmful NO*_x_* and unburned CH_4_ emissions from vehicle and power plant exhaust [1]. Metal-exchanged zeolite catalysts with relatively high catalytic activity over a wide temperature range are extensively studied in the CH_4_-SCR reaction. However, they suffer from poor hydrothermal stability, exhibiting a considerable activity decrease in the presence of high-temperature water vapor, especially those that are Al-rich and have large pores [2,3,4,5,6,7,8,9,10]. In the NH_3_-SCR field where ammonia is employed as a reducing agent, Cu-exchanged small-pore SSZ-13 zeolites (CHA topology) have been implemented as a new-generation catalyst in diesel after-treatment systems due to their high deNO*_x_* activity and good hydrothermal stability [11]. Recently, an alternative small-pore SSZ-39 zeolite (AEI topology), which has a different connection mode of neighboring double six-ring (*d6r*), demonstrated even better hydrothermal stability in NH_3_-SCR reactions [12]. Superior hydrothermal stability is a crucial requirement for a favorable CH_4_-SCR reaction, as it usually occurs at a relatively high temperature and the real exhaust always contains a certain amount of water vapor, exposing the catalyst to hydrothermal conditions during operation [13].

In our previous studies, we synthesized indium-exchanged zeolites for CH_4_-SCR and discovered that the framework type [14] and Si/Al ratio [15] of zeolites affected deNO*_x_* performance. The Si/Al ratio of a zeolite remarkably affects its acidity and stability. Zeolites with low Si/Al ratios possess abundant Brønsted acid sites (BASs, formed by protons compensating the negatively charged O atoms induced by the substitution of Si atoms by Al^IV^ atoms in the framework) serving as ion-exchange/active sites, but are more susceptible to dealumination under high-temperature hydrothermal conditions [12,16,17,18]. Steaming and/or acid leaching is an effective method for the selective extraction of Al atoms from the zeolite framework, enabling the convenient decrease of excessive acid densities and the modulation of Si/Al ratios [19]. Maintaining sufficiently enough exchangeable sites and zeolite integrity, however, needs to be considered in the dealumination treatment. Thus, carefully manipulating the mild dealumination of Al-rich zeolite is highly desirable so as to simultaneously achieve medium acidity and the preferred durability.

In the present work, commercial Al-rich H-SSZ-39 was dealuminated with oxalic acid (OA) and ion-exchanged with an indium nitrate aqueous solution to obtain In/H-SSZ-39(OA), exhibiting excellent deNO*_x_* activity in a dry CH_4_-SCR reaction. Although the Pristine SSZ-39 showed negligible deNO*_x_* activity after In exchange, the introduction of mild dealumination via weak acid etching prior to In exchange greatly improved the catalytic performance in the CH_4_-SCR reaction. Moreover, introducing a small amount of Co_3_O_4_ fine powder into the indium nitrate solution further enhanced the deNO*_x_* activity of the resulting In-Co_3_O_4_/H-SSZ-39(OA) catalysts. The efficacy of mild acid etching post-treatment and Co_3_O_4_ modification was tentatively illustrated based on comprehensive catalyst structure and reaction pathway analysis. ^27^Al and ^29^Si NMR, XRD, and N_2_ adsorption–desorption were conducted to characterize the zeolite framework structure changes. Microscopy, XPS, NH_3_-TPD, and NO-TPD were used to investigate the In/Co distributions, surface acid sites, and active intermediate N*_x_*O*_y_* species.

## 2. Results and Discussion

### 2.1. Catalytic Activity

#### 2.1.1. CH_4_-SCR Activity of In/H-SSZ-39(OA) Catalysts

**Figure 1:**
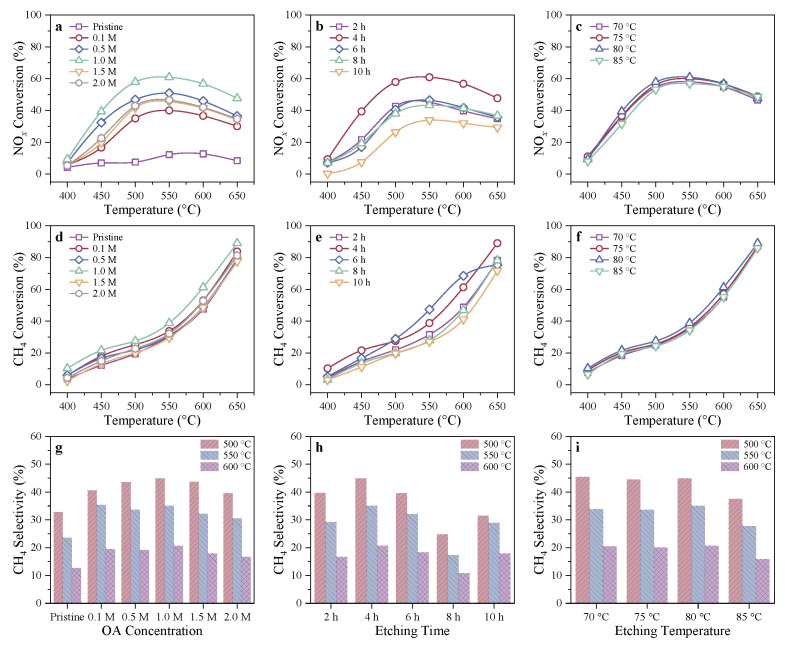
Effects of oxalic acid concentration, etching time, and etching temperature in acid etching post-treatment on the (**a**–**c**) NO*_x_* conversion, (**d**–**f**) CH_4_ conversion, and (**g**–**i**) CH_4_ selectivity of the resulting catalysts for dry CH_4_-SCR. Reaction conditions: [NO] = 400 ppm, [CH_4_] = 600 ppm, [O_2_] = 10 vol%, Ar balance, GHSV = 24,000 h^−1^.

The catalytic activity of In-exchanged Pristine H-SSZ-39 (In/H-SSZ-39) and a series of In/H-SSZ-39(OA) catalysts in CH_4_-SCR reaction was investigated. In-free Pristine H-SSZ-39 and H-SSZ-39(OA) samples were also tested for CH_4_-SCR reaction; however, they showed quite low deNO*_x_* activity (Figure S1a), indicating that the introduced indium species acted as the active component of the catalysts. As shown in Figure 1a, In/H-SSZ-39 without acid pretreatment exhibited very limited activity with <15% NO*_x_* conversion at 550 °C, while In/H-SSZ-39(OA) catalysts demonstrated considerably enhanced activity with >40% NO*_x_* conversion at 550 °C. Among them, the catalyst sample made from H-SSZ-39 etched with 1.0 M OA solution showed the highest NO*_x_* conversion (∼60% at 550 °C). Elevating the reaction temperature above 550 °C declined NO*_x_* conversion due to the non-selective oxidation of CH_4_, evidenced by the significantly enhanced CH_4_ conversion above 550 °C in Figure 1d–f and the dramatic difference between the CH_4_ selectivities at 550 °C and 600 °C in Figure 1g–i. Too low or high a concentration of OA led to an insignificant or excessive etching effect, detrimental to the CH_4_-SCR activity. Thus, the OA concentration in the SSZ-39 dealumination step was fixed at 1.0 M, and the effects of etching time and etching temperature were further investigated. As for the etching time, 4 h was found to be optimal (Figure 1b,e). The etching time of 2 h only slightly enhanced the deNO*_x_* activity of the catalyst. Extending the etching time to more than 4 h led to a gradual decrease in catalytic activity, and at the extreme of 10 h, the extensive etching yielded a low-efficiency catalyst that showed even more inferior performance than the In/H-SSZ-39 sample without etching pretreatment. A reasonable explanation could be that as etching proceeded, massive dealumination resulted in reduced exchangeable sites for In species in SSZ-39 zeolite and collapsed the zeolite framework. Compared to the significant effect of etching time, the etching temperature only have a minor effect on the catalytic activity, with the sample etched at 80 °C being slightly better than the others (Figure 1c,f).

#### 2.1.2. CH_4_-SCR Activity of In-Co_3_O_4_/H-SSZ-39(OA) Catalysts

**Figure 2:**
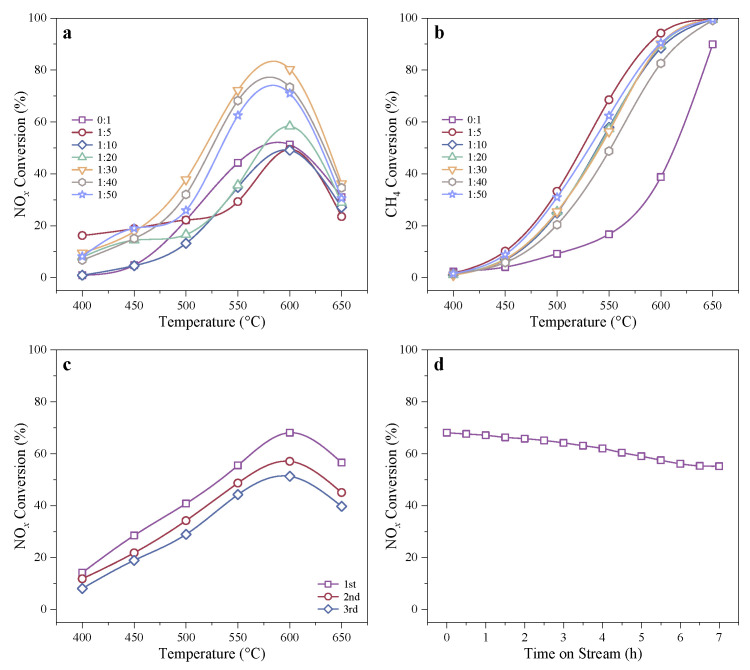
Effect of the Co_3_O_4_ to H-SSZ-39(OA) mass ratio on the (**a**) NO*_x_* conversion and (**b**) CH_4_ conversion of the resulting catalysts under wet conditions. Reaction conditions: [NO] = 400 ppm, [CH_4_] = 600 ppm, [O_2_] = 10 vol%, [H_2_O] = 5 vol%, Ar balance, GHSV = 24,000 h^−1^. (**c**) Recyclability test of In-Co_3_O_4_/H-SSZ-39(OA) under harsh H_2_O- and SO_2_-containing conditions. Reaction conditions: [NO] = 400 ppm, [CH_4_] = 600 ppm, [O_2_] = 10 vol%, [H_2_O] = 5 vol%, [SO_2_] = 50 ppm, Ar balance, GHSV = 12,000 h^−1^. (**d**) Stability test of In-Co_3_O_4_/H-SSZ-39(OA) under harsh H_2_O- and SO_2_-containing conditions. Reaction conditions: [NO] = 400 ppm, [CH_4_] = 600 ppm, [O_2_] = 10 vol%, [H_2_O] = 5 vol%, [SO_2_] = 50 ppm, Ar balance, GHSV = 12,000 h^−1^, *T* = 600 °C.

Previous studies have demonstrated that introducing small amounts of metal oxides (especially, Co_3_O_4_) into In-exchanged zeolite catalytic systems can improve CH_4_-SCR activity under harsh SO_2_- and H_2_O-containing conditions [20,21]. Therefore, small amounts of Co_3_O_4_ were dispersed during the In-exchange process on optimally acid-etched H-SSZ-39, with pretreatment using 1.0 M of OA at 80 °C for 4 h. The Co_3_O_4_-modifying amount for preparing In/H-SSZ-39(OA) catalysts (denoted as In-Co_3_O_4_/H-SSZ-39(OA)) in wet CH_4_-SCR reaction was investigated, as shown in the comparative catalytic activities (Figure 2a,b). Modification with trace amounts of Co_3_O_4_ (Co_3_O_4_ to zeolite mass ratio between 1/30–1/50) significantly boosted the highest NO*_x_* conversion to >70% and facilitated CH_4_ conversion even in the presence of 5 vol% H_2_O. The In-free sample of Co_3_O_4_/H-SSZ-39(OA) demonstrated boosted CH_4_ conversion compared to H-SSZ-39(OA) (Figure S1b), further proving that Co_3_O_4_ could promote CH_4_ conversion. Moreover, its negligible CH_4_-SCR activity (Figure S1a) signified that Co_3_O_4_ did not serve as a second active center responsible for NO*_x_* reduction but rather acted as a promoter. The highest NO*_x_* conversion over In/H-SSZ-39(OA) occurred at higher temperatures (∼600 °C), and the optimal amount of Co_3_O_4_ was determined to be 30:1, for this sample exhibited the highest NO*_x_* conversion of ∼83% (Figure 2a). The Co_3_O_4_-modified catalysts demonstrated higher NO*_x_* and CH_4_ conversion compared to Co_3_O_4_-free sample. Elevating the Co_3_O_4_ amount with a mass_zeolite_: ${\mathrm{mass}}_{\begin{array}{c} errortypeceCo3O4 \end{array}}$ ratio from 1:50 to 1:30 greatly enhanced NO*_x_* and CH_4_ conversions. A further increase in Co_3_O_4_ amount obviously lowered the NO*_x_* conversion. The CH_4_ conversions over In-Co_3_O_4_/H-SSZ-39(OA) catalysts under wet conditions were even higher than those of Co_3_O_4_-free ones under dry conditions (Figure 1d–f). The sample modified by the largest amount of Co_3_O_4_ (5:1) exhibited the highest CH_4_ conversion at 400–650 °C, but demonstrated the worst NO*_x_* conversion at a temperature range of 550–650 °C in CH_4_-SCR reaction, which was presumably related to the non-selective oxidation of methane catalyzed by excessive Co_3_O_4_ (Figure S2).

An In-Co_3_O_4_/H-SSZ-39(OA) catalyst prepared using the optimized OA etching conditions (0.1 M OA, 80 °C, 4 h) and Co_3_O_4_ dosage (Co_3_O_4_: H-SSZ-39(OA) mass ratio of 1:30) was tested under different CH_4_-SCR reaction conditions, as shown in Figure S3. Operation parameters including O_2_ concentration, CH_4_/NO ratio, H_2_O concentration, and GHSV all affected NO*_x_* and CH_4_ conversions as well as CH_4_ selectivity, with the effect of GHSV being most significant. Under a GHSV of 12,000 h^−1^, the highest NO*_x_* conversion of ∼88% occurred at 600 °C. The deNO*_x_* activity and CH_4_ selectivity roughly showed an increasing and then decreasing trend with O_2_ concentration, with the turning point occurring at an O_2_ concentration of 10 vol%. Higher CH_4_/NO ratios resulted in declined CH_4_ selectivity but slightly improved deNO*_x_* activity. The high concentration of water vapor adversely affected the catalyst, as evidenced by the continuously decreasing NO*_x_* conversion and CH_4_ selectivity with higher water vapor concentrations, while the CH_4_ conversion was largely unaffected. The tolerance to SO_2_ was also tested, and the In-Co_3_O_4_/H-SSZ-39(OA) catalyst maintained its high activity when the SO_2_ concentration was 50 ppm and 5 vol% water vapor was present (Figure S4). Therefore, even being operated under SO_2_- and H_2_O-containing conditions, the In-Co_3_O_4_/H-SSZ-39(OA) catalyst demonstrated excellent recyclability. The maximum NO*_x_* conversion of the catalyst in the third TPSR test was ∼50%, which was only ∼20% lower than that in the first test (Figure 2c). The catalyst also showed high stability, as shown in Figure 2d, the NO*_x_* conversion over the In-Co_3_O_4_/H-SSZ-39(OA) catalyst showed a slow downward trend for the first four hours and then gradually stabilized afterwards (∼55% at 600 °C). SO_2_ poisoning is mostly associated with the formation of sulphate species under oxidizing conditions [22], whereas H_2_O vapor usually led to the aggregation and sintering of active sites in the zeolite-based catalysts, forming weakly active or inactive metal oxide clusters [23,24].

### 2.2. Catalyst Characterization

#### 2.2.1. Microscopy

**Figure 3:**
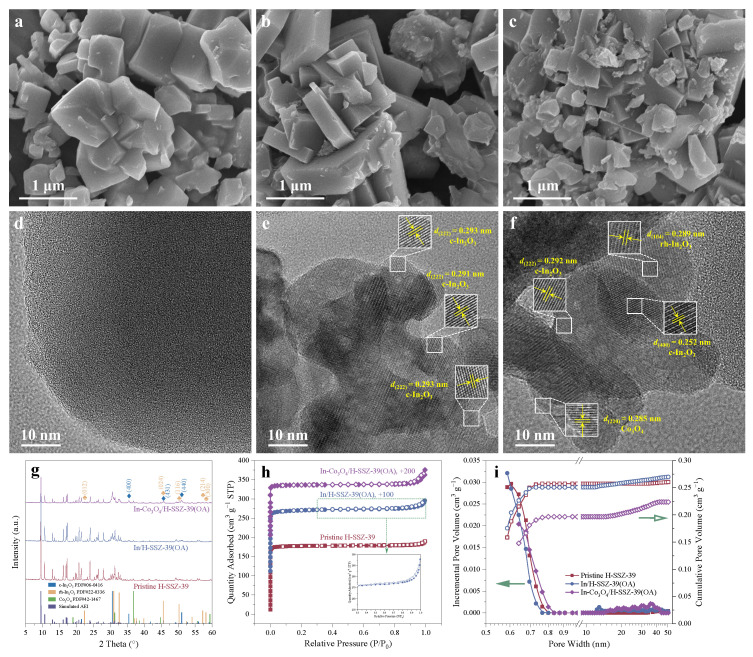
SEM images of (**a**) Pristine H-SSZ-39, (**b**) In/H-SSZ-39(OA), and (**c**) In-Co_3_O_4_/H-SSZ-39(OA). HRTEM images of (**d**) Pristine H-SSZ-39, (**e**) In/H-SSZ-39(OA), and (**f**) In-Co_3_O_4_/H-SSZ-39(OA). (**g**) PXRD patterns, (**h**) N_2_ adsorption–desorption isotherms, and (**i**) NLDFT PSD curves of samples.

In the SEM images of Pristine H-SSZ-39 (Figure 3a), cuboid particles with a mean size of ∼1 μm could be observed. Pristine SSZ-39 generally appeared as intact crystals with smooth surfaces and distinct edges. After acid etching and In exchange, the resulting In/H-SSZ-39(OA) exhibited mostly integrated crystals but partly with missing edges or surface depressions, suggesting that etching might start from the crystal periphery (Figure 3b). Elemental compositions determined by ICP-OES and elemental distributions of samples at different preparation stages were shown in Table 1 and Figures S5–S10. In species were uniformly distributed in In/H-SSZ-39(OA), without large indium oxide particles being observed (Figures S7 and S9); whereas for In-Co_3_O_4_/H-SSZ-39(OA), a few large indium and cobalt oxide particles were present (Figure 3c, S8, and S10). In a representative HRTEM image of In/H-SSZ-39(OA) (Figure 3e), some nanoparticles attached to the zeolite surface with a lattice spacing of ∼0.293 nm assigned to the cubic In_2_O_3_ (c-In_2_O_3_) (222) crystal plane could be observed. Meanwhile, some mesopores appeared in the In/H-SSZ-39(OA) zeolite (Figure S11b), which contrasted with the Pristine SSZ-39 (Figure 3d and S11a). In-Co_3_O_4_/H-SSZ-39(OA) contained apparently broken crystal fragments with irregular shapes and a more obvious mesopore structure (Figure 3f and S11c). As shown in Figure 3f, c-In_2_O_3_, rhombohedral In_2_O_3_ (rh-In_2_O_3_), and Co_3_O_4_ nanoparticles were distributed on In-Co_3_O_4_/H-SSZ-39(OA), as confirmed by lattice fringes attributed to c-In_2_O_3_ (222) (*d* = 0.292 nm), c-In_2_O_3_ (400) (*d* = 0.252 nm), rh-In_2_O_3_ (104) (*d* = 0.289 nm), and Co_3_O_4_ (220) (*d* = 0.285 nm). The zeolite framework, with lattice fringes (*d* = 0.911 nm) assigned to AEI (110) or (002) crystal planes were the bulk phase, as shown in Figure S11c.

#### 2.2.2. Crystalline Properties

As observed from Figure 3g, PXRD patterns demonstrated that Pristine H-SSZ-39 was a phase-pure AEI zeolite that matched well with the simulated structure. After acid leaching, the diffraction peaks slightly shifted toward higher 2θ values, suggesting a lattice contraction as a result of a change in the chemical composition, that was an extraction of Al from the unit cell of SSZ-39 (Figure S12a). Accordingly, the relative crystallinity slightly decreased to 92.5% after OA leaching. Indium exchange introduced In atoms that are larger than the host atoms, leading to lattice expansion, and thus the diffraction peaks slightly shifted toward lower 2θ values. Modification with Co_3_O_4_ did not result in any shift in the diffraction peaks, signifying that Co_3_O_4_ might not incorporate into the interior of SSZ-39 zeolite. It had been reported that the migration of In species would have an impact on the zeolite channel structure [25], which might contribute to the decrease in relative crystallinity after In exchange (Table 1). In species (e.g., In_2_O_3_) were undetectable by XRD for In/H-SSZ-39(OA), likely because of their low loading, small size, and highly dispersed distribution, consistent with the EDS mapping results (Figures S7 and S9). Distinct characteristic diffraction peaks of c-In_2_O_3_ and rh-In_2_O_3_ could be observed from the PXRD pattern of In-Co_3_O_4_/H-SSZ-39(OA) (Figure 3g, S12c,d, and S13b), which might be attributed to the reduced exchangeable sites in the partly amorphized structure of this sample (relative crystallinity = 33.4%, Table 1). In species that exceeded the exchange capacity of zeolites with reduced crystallinity formed extra-zeolite In_2_O_3_ particles during calcination, which were typically inactive in the catalyzing CH_4_-SCR reaction [26,27]. Moreover, diffraction peaks attributed to Co_3_O_4_ were found for In-Co_3_O_4_/H-SSZ-39(OA) in Figure S12b, even though some of the diffraction peaks of multiple components overlapped each other and those from the Co_3_O_4_ component were very weak. No detectable indium oxides and cobalt oxides by XRD other than In_2_O_3_ and Co_3_O_4_ were present (Figure S13), which corresponded well with HRTEM observation.

#### 2.2.3. Textural Properties

The samples were characterized by N_2_ adsorption–desorption isotherm measurements at 77 K (Figure 3h), and their textural properties including non-local density functional theory (NLDFT) pore size distributions (PSD) are shown in Table 1 and Figure 3i. Pristine H-SSZ-39 exhibited a typical type I isotherm, characterized by a sharp rise in N_2_ adsorption at low pressure (*p*/*p*_0_ < 0.01) followed by a saturation plateau, indicating the predominance of microporosity. No obvious hysteresis loop was observed, corresponding to its negligible mesoporosity (Table 1 and Figure 3i). The condensation of N_2_ molecules in interparticle voids contributed to a minor increase in adsorption at *p*/*p*_0_ ≈ 1.0. Acid etching and In exchange slightly decreased the microporosity while increasing the mesoporosity, indicating that a small number of neighboring micropores merged to form mesopores during Al extraction and In migration. In/H-SSZ-39(OA) had a similar type I isotherm but a more pronounced condensation of N_2_ molecules, which might be associated the increased mesoporosity in the zeolite (Figure 3i inset and Table 1). This phenomenon was most noticeable in In-Co_3_O_4_/H-SSZ-39(OA), and further amorphorized zeolite fragments might also make a contribution, consistent with the microscopy observation and PXRD measurement. In addition, In-Co_3_O_4_/H-SSZ-39(OA) had the lowest low-pressure N_2_ adsorption capacity, corresponding to its smallest micropore volume (0.202 cm^3^ g^−1^). As shown in Figure 3i, the mesopores in In/H-SSZ-39(OA) and In-Co_3_O_4_/H-SSZ-39(OA) were mainly distributed in 20–50 nm. The constructed hierarchical structure might contribute to the enhanced catalytic activity through enhanced mass transfer.

#### 2.2.4. Coordination Environment of T-Atoms

**Figure 4:**
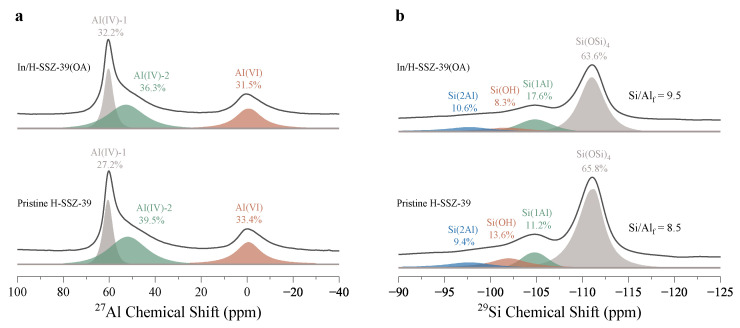
(**a**) ^27^Al MAS SSNMR and (**b**) ^29^Si MAS SSNMR of Pristine H-SSZ-39 and In/H-SSZ-39(OA).

Figure 4a depicted ^27^Al MAS SSNMR spectra of Pristine H-SSZ-39 and In/H-SSZ-39(OA). For Pristine H-SSZ-39, the sharp signal at ∼60 ppm was associated with tetrahedra framework Al (FAl) sites, designated as Al(IV)-1. In addition, a broad peak centered at ∼52 ppm was attributed to the partially coordinated FAl atoms with hydroxyl groups (${\left(\mathrm{SiO}\right)}_{4-n}$−Al(OH)n, *n* = 1–3), commonly known as Al(IV)-2, which usually resulted from synthesis and post-treatment processes [28,29,30,31]. Another signal at ∼0 ppm was ascribed to octahedrally coordinated Al (denoted as Al(VI)), namely extra-framework Al (EFAl) [32]. Acid etching dramatically changed the Al coordination environment of H-SSZ-39(OA), with Al(IV)-1 becoming the dominant component (∼48.7%) and the proportions of Al(IV)-2 and Al(VI) atoms decreased (Figure S14a). This provided direct evidence for the preferential interaction of OA with Al−OH groups and EFAl. For In/H-SSZ-39(OA), Al(IV)-2 again became the dominant component (∼36.3%), with a concomitant increase in Al(VI) and a decrease in Al(IV)-1. The migration of In species likely affected the zeolite pore structure that was accompanied by FAl-to-EFAl conversion [25]. In the ^29^Si MAS SSNMR (Figure 4b), the signals at −111, −105, and −99 ppm could be assigned to Si(0Al), Si(1Al), and Si(2Al), respectively. In addition, a signal positioned between Si(1Al) and Si(2Al) was identified as Si−OH groups [31]. The ^29^Si MAS SSNMR spectra were used to estimate the Si/Al ratio in the zeolite framework (abbreviated as Si/Al_f_) [33] according to Equation (1). The calculated Si/Al_f_ followed a sequence of 13.8 for H-SSZ-39(OA) > 9.46 for In/H-SSZ-39(OA) > 8.5 for Pristine H-SSZ-39 (Figure 4b and S14b). The framework Si/Al_f_ ratios were higher compared to their bulk Si/Al ratios, also signifying the presence of EFAl, consistent with the ^27^Al MAS NMR measurements. The increased Si/Al_f_ ratio after acid etching indicated the selective extraction of FAl by OA; whereas the decreased Si/Al_f_ ratio after In exchange could be related to the migration of In species.

#### 2.2.5. Surface Chemical State

**Figure 5:**
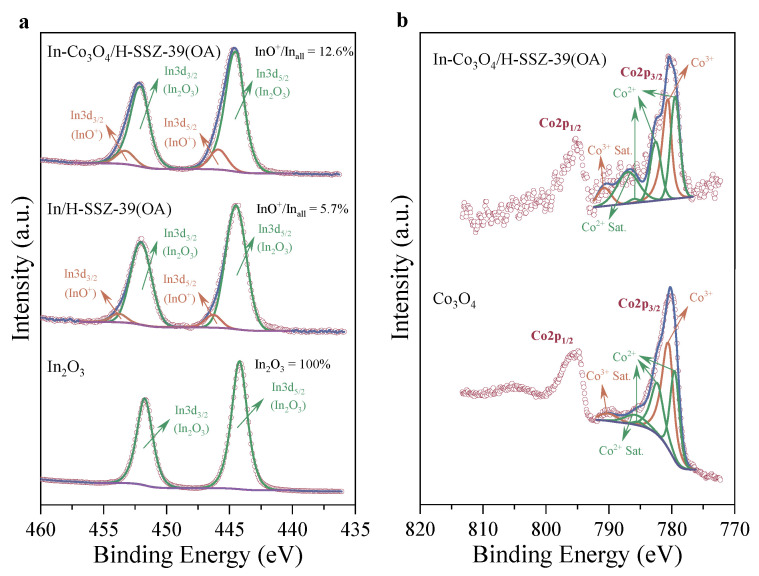
High-resolution XPS spectra of (**a**) In 3d region and (**b**) Co 2p region.

The chemical states of the catalyst surface components were examined using XPS. The In content on the In/H-SSZ-39(OA) surface measured by XPS (∼5.98 wt%, Figure S15a) was higher than that in the bulk phase determined by ICP (∼4.40 wt%, Table 1). For In-Co_3_O_4_/H-SSZ-39(OA), the surface enrichment of In and Co was also observed (Figure S15b and Table 1), consistent with the formation of In/Co oxides on the surface as observed by HRTEM. As shown in Figure 5a, In 3d spectral peaks of both In/H-SSZ-39(OA) and In-Co_3_O_4_/H-SSZ-39(OA) broadened with respect to those of reference In_2_O_3_, indicating the presence of a second In species that was commonly recognized as InO^+^ [25,34,35]. It was reported that InO^+^ species were the principal active centers in In-exchanged zeolitic CH_4_-SCR catalysts responsible for CH_4_ activation and active N*_x_*O*_y_* formation [15,25,36]. The percentage of InO^+^ species (InO^+^/Inall) in the In/H-SSZ-39(OA) catalyst was 5.7%. With Co_3_O_4_ modification, the percentage of InO^+^ species in In-Co_3_O_4_/H-SSZ-39(OA) increased to 12.6%, which could contribute to the high CH_4_-SCR activity of the In-Co_3_O_4_/H-SSZ-39(OA) catalyst. Figure 5b depicted the high-resolution Co 2p spectra of reference Co_3_O_4_ and In-Co_3_O_4_/H-SSZ-39(OA). After deconvolution, it could be concluded that Co(II) oxide and Co(III) oxide coexisted on the In-Co_3_O_4_/H-SSZ-39(OA) surface according to the distinguishable characteristic satellite peaks of Co^2^+ (BE ≈ 787.0 eV) and Co^3^+ (BE ≈ 790.9 eV). Additionally, no detectable cobalt oxides other than Co_3_O_4_ were observed in the XRD analysis (Figure S13a), further indicating the presence of Co_3_O_4_ on the In-Co_3_O_4_/H-SSZ-39(OA) surface.

#### 2.2.6. Surface Acidity

**Figure 6:**
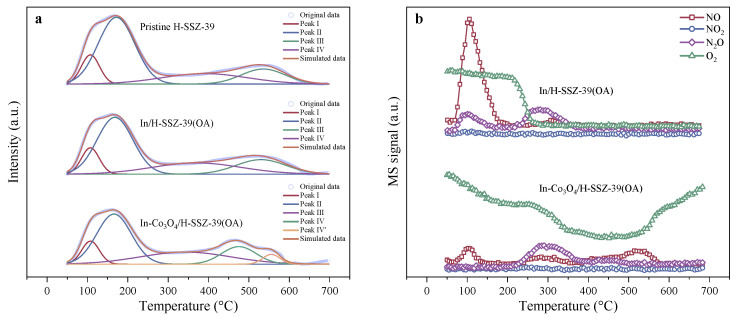
(**a**) NH_3_-TPD profiles and (**b**) NO-TPD profiles of samples.

NH_3_-TPD measurements were performed to assess the quantity and strength of surface acid sites of samples. As depicted in Figure 6a, the NH_3_-TPD profile of the Pristine H-SSZ-39 could be deconvoluted with the Gaussian algorithm to four distinct NH_3_ desorption peaks within the temperature range of 50–700 °C. Peak I (∼107 °C) was assigned to surface hydroxyl groups, such as Si−OH and Al−OH [25]. Peak II (∼172 °C) and Peak III (∼390 °C) were associated with the desorption of NH_3_ bound to weak and strong Lewis acid sites (LAS, such as EFAl), respectively. Peak IV (∼536 °C) corresponded to NH_3_ desorption from strong Brønsted acid sites (BAS, Si−OH−Al) [9,33,37,38]. In/H-SSZ-39(OA) exhibited a similar NH_3_-TPD profile, but the total acid quantity (1.276 mmol g^−1^) was reduced compared to H-SSZ-39 (1.366 mmol g^−1^), as presented in Table 2. Specifically, the quantity of weak LAS and strong BAS decreased, consistent with the expected results of acid etching [39]. The acid-etching dealumination might preferentially take place from EFAl compared to FAl, considering the presence of FAl-to-EFAl conversion during dealumination as well as the consumption of BAS during In exchange, which was consistent with the ^27^Al MAS SSNMR results. On the other hand, the introduced In species apparently contributed to the increased strong LAS density. For In-Co_3_O_4_/H-SSZ-39(OA), the similar NH_3_ desorption peaks could be observed at low temperatures (∼108 °C and ∼166 °C) with further decreased intensity, which might be associated with the partial coverage of the zeolite surface by In_2_O_3_ and Co_3_O_4_ nanoparticles. The peak assigned to NH_3_ desorption from BAS, however, was split into two peaks (denoted as Peak IV and Peak IV’) in In-Co_3_O_4_/H-SSZ-39(OA); one was at ∼475 °C and the other one was at ∼556 °C. It was reported that the NH_3_ desorption peak for pure Co_3_O_4_ was lower than 220 °C with a much smaller NH_3_ desorption amount than those from parent zeolites or Co/zeolites [40]. The strong NH_3_ desorption at ∼556 °C over In-Co_3_O_4_/H-SSZ-39(OA) suggested a probable synergistic interaction between Co species and the support acid sites. Similar phenomena had been observed in Co/Beta and Co/ZSM-5 zeolite [41].

#### 2.2.7. N*_x_*O*_y_* Intermediates during Reaction

NO could be adsorbed on H-SSZ-39 in the form of N*_x_*O*_y_* species via the interaction with acidic hydroxyl groups in the zeolite [6]. Thus, NO-TPD was an effective tool to provide insights into possible surface N*_x_*O*_y_* species and their stability during SCR reactions over catalysts. As shown in Figure 6b, the vast majority of N*_x_*O*_y_* species escape from In/H-SSZ-39(OA) below 350 °C, with NO and N_2_O detected as major desorption and/or decomposition products; whereas at higher temperatures, only a weak NO desorption peak centered at ∼580 °C was observed. Obviously, these weakly bound N*_x_*O*_y_* species with low decomposition temperatures could not persist at the active temperature (400–650 °C) for the CH_4_-SCR reaction. Therefore, lacking available intermediate species, the CH_4_-SCR activity of In/H-SSZ-39(OA) was relatively low. On the contrary, for In-Co_3_O_4_/H-SSZ-39(OA), both a weak NO desorption peak at ∼104 °C and a strong NO desorption peak at ∼525 °C were observed. In addition, N_2_O was detected as a desorption and/or decomposition product at high temperatures, indicating that it might also be an available active N*_x_*O*_y_* species to serve as intermediates for CH_4_-SCR. It was tentatively concluded that a small amount of Co_3_O_4_ rendered In-Co_3_O_4_/H-SSZ-39(OA) with more strong adsorption sites to enrich stable N*_x_*O*_y_* species reserves at high temperatures, thus boosting the CH_4_-SCR activity of the catalyst.

## 3. Materials and Methods

### 3.1. Acid Etching

One gram of commercial H-SSZ-39 zeolite (Si/Al = 4.72, Dalian Huayizhongxin New Material Co., Ltd., Dalian, China) was ultrasonically dispersed in 25 mL of 1 M oxalic acid (oxalic acid dihydrate, AR, Damao Chemical Reagent Factory, Tianjin, China) aqueous solution. After stirring at 80 °C for 4 h, the etched H-SSZ-39 was recovered by suction filtration and washed with ultrapure water several times until the pH of the filtrate was approximately neutral. The filter cake was dried at 80 °C overnight followed by grinding and calcination in a muffle furnace at 500 °C for 3 h to obtain OA-etched H-SSZ-39, denoted as H-SSZ-39(OA).

### 3.2. Indium Exchange

A total of 0.3 g of H-SSZ-39(OA) zeolites were dispersed in 10 mL of 0.066 M indium nitrate (indium nitrate hydrate, 99.99% metals basis, Shanghai Aladdin Biochemical Technology Co., Ltd., Shanghai, China) aqueous solution and the suspension was stirred at 85 °C for 8 h. In-containing H-SSZ-39(OA) was recovered by centrifugation and washed twice with ultrapure water. After drying at 80 °C overnight, the sample was calcined in a muffle furnace at 500 °C for 3 h to obtain In/H-SSZ-39(OA). In-Co_3_O_4_/H-SSZ-39(OA) was prepared by the same process as that for In/H-SSZ-39(OA), except that a certain amount of Co_3_O_4_ fine powder (cobalt oxide, 99.9% metals basis, Shanghai Aladdin Biochemical Technology Co., Ltd., Shanghai, China) was suspended in the indium nitrate aqueous solution with the Co_3_O_4_: H-SSZ-39(OA) mass ratios of 1:5, 1:10, 1:20, 1: 30, 1:40, and 1:50.

### 3.3. Catalyst Characterizations

The microstructures of samples were observed with a JEOL JSM-7800F (Tokyo, Japan) field emission scanning electron microscope (FESEM) equipped with X-MaxN Falcon (Oxford Instruments, Abingdon, UK) energy dispersive X-ray spectroscopy (EDS) at 5 kV (15 kV for EDS mapping measurement) and a Talos F200X G2 (Thermo Fisher Scientific, Norristown, PA, USA) scanning transmission electron microscope ((S)TEM) equipped with Super-X G2 (Thermo Fisher Scientific) EDS at 200 kV. In, Co contents, and bulk Si/Al ratios of samples were measured using inductively coupled plasma optical emission spectroscopy (ICP-OES) on an Agilent 720ES (Santa Clara, CA, USA) instrument. N_2_ adsorption–desorption isotherms measured at 77 K (Micromeritics ASAP2460, Norcross, GA, USA) were used to analyze specific surface areas and pore size distributions of the catalysts. The samples were degassed at 300 °C for 8 h before measurement. Powder X-ray diffraction (PXRD) patterns of samples were recorded by a Rigaku D/Max-2200 PC diffractometer (Tokyo, Japan) in the diffraction angle range of 2θ = 5–60° with Cu Kα radiation (λ = 1.5418 Å) at 40 kV and 50 mA. X-ray photoelectron spectra (XPS) were recorded by a Thermo Scientific K-Alpha instrument (America) with a monochromatic Al Kα (1486.6 eV) as an X-ray source. The binding energy values were calibrated using the C 1s peak at 284.8 eV for adventitious carbon. Solid-state nuclear magnetic resonance (SSNMR) experiments were performed on a Bruker 400M spectrometer (Bremen, Germany). The deconvolutions of spectra were performed with the ssNake v1.4 software [42]. The single-pulse ^29^Si magic angle spinning (MAS) SSNMR spectra were acquired on a 7 mm probe with a spinning rate of 5 kHz, a pulse width of 5.6 μs, a relaxation delay of 5 s, and 256 scans. The ^29^Si chemical shifts were referenced to tetramethylsilane (TMS) at 0 ppm, and the framework Si/Al_f_ ratios were estimated using the following Equation (1).

(1) $$\mathrm{Si}/{\mathrm{Al}}_{f}=\frac{\sum_{n=0}^{4}I_{\mathrm{Si}(n\mathrm{Al})}}{\sum_{n=0}^{4}0.25n\left[I_{\mathrm{Si}(n\mathrm{Al})}\right]}$$

where $I_{\mathrm{Si}(n\mathrm{Al})}$ was the signal intensity of Si with different numbers of incorporated Al atoms (*n* = 0–4) in its first Si(OT)_4_ (T representing framework Si and Al atoms) coordination shell. The single-pulse ^27^Al MAS SSNMR were acquired on a 4 mm probe with a spinning rate of 10 kHz, a pulse width of 1.48 μs, a relaxation delay of 0.1 s, and 2048 scans. The ^27^Al chemical shifts were referenced to NaAlO_2_ at 65 ppm. Ammonia temperature-programmed desorption (NH_3_-TPD) was carried out on a Micromeritics AutoChem II 2920 chemisorption analyzer (America). A total of 100 mg of sample was loaded into a quartz reactor and pretreated in air at 500 °C for 60 min. After cooling to 50 °C, 10% NH_3_/He was introduced at a flow rate of 50 mL min^−1^ for 60 min to saturate the sample with NH_3_, followed by introducing He flow as purge gas. The NH_3_-TPD profile was then recorded by a thermal conductivity detector (TCD) across the temperature range of 50 °C to 700 °C at a ramping rate of 10 °C min^−1^. Nitric oxide temperature-programmed desorption (NO-TPD) analysis was performed on a TP-5080-B instrument (Tianjin, China) equipped with a Hiden DECRA mass spectrometer (Warrington, UK). A total of 100 mg of sample was loaded into a quartz reactor and pretreated in a He stream (30 mL min^−1^) at 500 °C for 60 min. After cooling to 50 °C, the He flow was switched to a 10% NO/He gas mixture at a flow rate of 30 mL min^−1^. The weakly physically adsorbed NO was removed by purging with He gas flow (30 mL min^−1^) for 60 min before programmed warming at 10 °C min^−1^. The following mass fragments sensible to the system perturbation were monitored on-line in the temperature range of 50–660 °C: NO (*m*/*z* = 30), O_2_ (*m*/*z* = 32), N_2_O (*m*/*z* = 44), and NO_2_ (*m*/*z* = 46).

### 3.4. Catalytic Activity Test

CH_4_-SCR activity was tested at atmospheric pressure using a certain mass of 40–60 mesh catalyst loaded in a fixed-bed quartz reactor [21]. A gas mixture composed of 400 ppm CH_4_, 600 ppm NO, 10 vol% O_2_, and 5 vol% H_2_O (optional) with an Ar balance at a flow rate of 100 mL min^−1^ was introduced into the reactor, corresponding to a gas hourly space velocity (GHSV) of ∼24,000 h^−1^ for 0.1 g of catalyst. The concentrations of NO*_x_* were monitored by a nitrogen oxide analyzer (Teledyne Model T200H, Thousand Oaks, CA, USA), while CH_4_, CO, and CO_2_ concentrations were analyzed by a gas chromatograph (Fuli GC9790II, Taizhou, China) equipped with a Porapak-Q column (Agilent, America) and a flame ionization detector (FID). The NO*_x_* and CH_4_ conversions as well as CH_4_ selectivity were calculated using Equations (2)–(4), respectively.

(2) $${\mathrm{NO}}_{x}\;\mathrm{conversion}\;\left(\%\right)=\frac{{\left[{\mathrm{NO}}_{x}\right]}_{\mathrm{in}}-{\left[{\mathrm{NO}}_{x}\right]}_{\mathrm{out}}}{{\left[{\mathrm{NO}}_{x}\right]}_{\mathrm{in}}}\times 100\%$$

(3) $${\mathrm{CH}}_{4}\;\mathrm{conversion}\;\left(\%\right)=\frac{{\left[{\mathrm{CH}}_{4}\right]}_{\mathrm{in}}-{\left[{\mathrm{CH}}_{4}\right]}_{\mathrm{out}}}{{\left[{\mathrm{CH}}_{4}\right]}_{\mathrm{in}}}\times 100\%$$

(4) $${\mathrm{CH}}_{4}\;\mathrm{selectivity}\;\left(\%\right)=0.5\times \frac{{\left[{\mathrm{NO}}_{x}\right]}_{\mathrm{in}}-{\left[{\mathrm{NO}}_{x}\right]}_{\mathrm{out}}}{{\left[{\mathrm{CH}}_{4}\right]}_{\mathrm{in}}-{\left[{\mathrm{CH}}_{4}\right]}_{\mathrm{out}}}\times 100\%$$

where NO*_x_* represents NO and NO_2_; the subscripts “in” and “out” represent inlet and outlet, respectively.

## 4. Conclusions

In summary, In-Co_3_O_4_/H-SSZ-39(OA) has been successfully constructed and applied as a robust catalyst in CH_4_-SCR reaction under harsh conditions. Specifically, the NO conversion of ∼80% could be achieved at ∼600 °C under a GHSV of 24,000 h^−1^ and in the presence of 5 vol% H_2_O, significantly outperforming the In/H-SSZ-39 without acid etching pretreatment and the In/H-SSZ-39(OA) without Co_3_O_4_ modification. Moreover, good stability was achieved on In-Co_3_O_4_/H-SSZ-39(OA) and <15% activity loss could be observed within 7 h at 600 °C. The mild acid leaching with OA delicately tuned the Si/Al ratio of SSZ-39 zeolite. During this process, OA was found to preferentially interact with Al−OH and EFAl, with some mesopores introduced while maintaining relative high crystallinity. A small amount of Co_3_O_4_ greatly improved the catalytic activity of the catalyst despite causing the severely decreased the crystallinity of SSZ-39 zeolite. Co_3_O_4_ could promote CH_4_ conversion and render a much higher storage of stable N*_x_*O*_y_* species available at high temperatures. 

## Figures and Tables

**Table 1:** Physicochemical parameters of Pristine H-SSZ-39, In/H-SSZ-39(OA), and In-Co_3_O_4_/H-SSZ-39(OA).

Sample	Relative Crystallinity *^a^* (%)	*S*_BET_ *^b^* (m^2^ g^−1^)	*V*_tot_ *^c^* (cm^3^ g^−1^)	*V*_micro_ *^d^* (cm^3^ g^−1^)	*V*_meso_ *^e^* (cm^3^ g^−1^)	Si/Al Bulk *^f^*	In Content *^f^* (wt%)	Co Content *^f^* (wt%)
Pristine H-SSZ-39	100.0	746.7	0.293	0.278	0.015	4.72	/	/
In/H-SSZ-39(OA)	72.0	699.5	0.303	0.242	0.060	6.79	4.40	/
In-Co_3_O_4_/H-SSZ-39(OA)	33.4	566.6	0.272	0.202	0.070	6.86	7.17	1.14

*^a^* Calculated from the sum of the integral areas of diffraction peaks ascribed to (111), (200), (113), (310), (132), (133), and (025) crystal planes. *^b^* Calculated by the Brunauer–Emmett–Teller (BET) model. *^c^* Calculated from the adsorption amount at a relative pressure (P/P_0_) close to 1. *^d^* Calculated using the *t*-plot method. *^e^* Calculated as the difference between *V*_tot_ and *V*_micro_. *^f^* Determined by ICP-OES.

**Table 2:** The strength and quantity of surface acid sites of samples based on NH_3_-TPD measurements.

Sample	Peak I		Peak II		Peak III		Peak IV	
	*T* (°C)	*Q* (mmol g^−1^)	*T* (°C)	*Q* (mmol g^−1^)	*T* (°C)	*Q* (mmol g^−1^)	*T* (°C)	*Q* (mmol g^−1^)
Pristine H-SSZ-39	107.4	0.160	171.8	0.737	390.4	0.199	535.7	0.269
In/H-SSZ-39(OA)	107.2	0.141	168.1	0.615	370.3	0.301	530.1	0.219
In-Co_3_O_4_/H-SSZ-39(OA)	107.6	0.127	165.9	0.541	343.0	0.340	475.1 + 555.6	0.190 + 0.053

## Data Availability

Dataset available on request from the authors.

## Supplementary Materials

The following supporting information can be downloaded at: https://www.mdpi.com/article/10.3390/molecules29163747/s1, Figure S1: The CH_4_-SCR deNO*_x_* performance of Pristine H-SSZ-39, H-SSZ-39(OA), and Co_3_O_4_/H-SSZ-39(OA) under dry conditions; Figure S2: The effect of the Co_3_O_4_ to H-SSZ-39(OA) mass ratio on the CH_4_ selectivity of the resulting catalysts under wet conditions; Figure S3: The CH_4_-SCR deNO*_x_* performance of In-Co_3_O_4_/H-SSZ-39(OA) at different O_2_ concentrations, CH_4_/NO ratios, water vapor concentrations and GHSVs; Figure S4: The CH_4_-SCR deNO*_x_* performance of In-Co_3_O_4_/H-SSZ-39(OA) at different SO_2_ concentrations and in the presence of 5 vol% H_2_O; Figures S5–S8: SEM-EDS mapping of Pristine H-SSZ-39, H-SSZ-39(OA), In/H-SSZ-39(OA), and In-Co_3_O_4_/H-SSZ-39(OA); Figures S9 and S10: HAADF-STEM and elemental mapping images of In/H-SSZ-39(OA) and In-Co_3_O_4_/H-SSZ-39(OA); Figure S11: HRTEM images of Pristine H-SSZ-39, In/H-SSZ-39(OA), and In-Co_3_O_4_/H-SSZ-39(OA); Figure S12: Locally enlarged PXRD patterns in the ranges of 7.5–15.0°, 18.0–20.0°, 34.0–36.0°, and 57.0–59.0° of Pristine H-SSZ-39, H-SSZ-39(OA), In/H-SSZ-39(OA), and In-Co_3_O_4_/H-SSZ-39(OA); Figure S13: PXRD patterns of In-Co_3_O_4_/H-SSZ-39(OA) in comparison with standard PXRD patterns of cobalt oxides and indium oxides; Figure S14: ^27^Al MAS SSNMR and ^29^Si MAS SSNMR spectra of H-SSZ-39(OA); Figure S15: XPS survey spectra and corresponding surface element contents of In/H-SSZ-39(OA) and In-Co_3_O_4_/H-SSZ-39(OA).

## References

[1] Xu Y., Wang X., Qin M., Li Q. (2022). Selective catalytic reduction of NO_x_ with CH_4_ over zeolite catalysts: Research progress, challenges and perspectives. J. Environ. Chem. Eng..

[2] Li Y., Armor J. (1992). Catalytic reduction of nitrogen oxides with methane in the presence of excess oxygen. Appl. Catal. B.

[3] Ramallo-López J., Requejo F., Gutierrez L., Miró E. (2001). EXAFS, TDPAC and TPR characterization of PtInFerrierite: The role of surface species in the SCR of NO_x_ with CH_4_. Appl. Catal. B.

[4] Bustamante F., Córdoba F., Yates M., Montes de Correa C. (2002). The promotion of cobalt mordenite by palladium for the lean CH_4_-SCR of NO_x_ in moist streams. Appl. Catal. A.

[5] Anunziata O.A., Beltramone A.R., Requejo F.G. (2007). In-containing BEA zeolite for selective catalytic reduction of NO_x_: Part I: Synthesis, characterization and catalytic activity. J. Mol. Catal. A Chem..

[6] Yang J., Chang Y., Dai W., Wu G., Guan N., Li L. (2018). Ru-In/H-SSZ-13 for the selective reduction of nitric oxide by methane: Insights from temperature-programmed desorption studies. Appl. Catal. B.

[7] Yang J., Chang Y., Dai W., Wu G., Guan N., Li L. (2018). Bimetallic Cr-In/H-SSZ-13 for selective catalytic reduction of nitric oxide by methane. Chin. J. Catal..

[8] Zhao J., Dong L., Wang Y., Zhang J., Zhu R., Li C., Hong M. (2022). Amino-acid modulated hierarchical In/H-Beta zeolites for selective catalytic reduction of NO with CH_4_ in the presence of H_2_O and SO_2_. Nanoscale.

[9] Wang C., Lv G., Li Y., Liu Y., Song C. (2024). Selective catalytic reduction of NO_X_ with CH_4_ over In/SSZ-13 zeolites: The enhancement of high-temperature catalytic activity by Ce modification. J. Environ. Chem. Eng..

[10] Wang X., Ge W., Liu Y., Miao B., Qin M., Ji C., Li Q. (2024). Boosting the activity of In-H-SSZ-13 via tuning acid sites for synergistic abatement of NO_x_ and CH_4_. Fuel.

[11] Jabłońska M. (2022). Review of the application of Cu-containing SSZ-13 in NH_3_-SCR-DeNO*x* and NH_3_-SCO. RSC Adv..

[12] Shan Y., Shan W., Shi X., Du J., Yu Y., He H. (2020). A comparative study of the activity and hydrothermal stability of Al-rich Cu-SSZ-39 and Cu-SSZ-13. Appl. Catal. B.

[13] Gui R., Yan Q., Xue T., Gao Y., Li Y., Zhu T., Wang Q. (2022). The promoting/inhibiting effect of water vapor on the selective catalytic reduction of NO_x_. J. Hazard. Mater..

[14] Zhao J., Zhang G., He J., Wen Z., Li Z., Gu T., Ding R., Zhu Y., Zhu R. (2020). Effect of preparation and reaction conditions on the performance of In/H-Beta for selective catalytic reduction of NO_x_ with CH_4_. Chemosphere.

[15] Zhao J., Li Z., Zhu R., Zhang J., Ding R., Wen Z., Zhu Y., Zhang G., Chen B. (2021). Mechanism of the selective catalytic reduction of NO_x_ with CH_4_ on In/H-beta. Catal. Sci. Technol..

[16] Sun Y., Fu Y., Shan Y., Du J., Liu Z., Gao M., Shi X., He G., Xue S., Han X. (2022). Si/Al Ratio Determines the SCR Performance of Cu-SSZ-13 Catalysts in the Presence of NO_2_. Environ. Sci. Technol..

[17] Wang Z., Jiang Y., Lafon O., Trébosc J., Duk Kim K., Stampfl C., Baiker A., Amoureux J.P., Huang J. (2016). Brønsted acid sites based on penta-coordinated aluminum species. Nat. Commun..

[18] Liu C., Malta G., Kubota H., Toyao T., Maeno Z., Shimizu K.i. (2021). Mechanism of NH_3_–Selective Catalytic Reduction (SCR) of NO/NO_2_ (Fast SCR) over Cu-CHA Zeolites Studied by *In Situ/Operando* Infrared Spectroscopy and Density Functional Theory. J. Phys. Chem. C.

[19] Chen G., Zhao N., Chen Y., Zhao J., Zhu R., Hong M. (2024). Recent advances in synthetic strategies and physicochemical modifications of SSZ-13 zeolites: A review. Mater. Today Catal..

[20] Zhao J., Wen Z., Zhu R., Li Z., Ding R., Zhu Y., Gu T., Yang R., Zhu Z. (2020). In/H-Beta modified by Co_3_O_4_ and its superior performance in the presence of H_2_O and SO_2_ for selective catalytic reduction of NO_x_ with CH_4_. Chem. Eng. J. Adv..

[21] Zhao M., Zhao J., Ding R., Zhu R., Li H., Li Z., Zhang J., Zhu Y., Li H. (2022). Insights into the superior resistance of In-Co_3_O_4_-Ga_2_O_3_/H-Beta to SO_2_ and H_2_O in the selective catalytic reduction of NO_x_ by CH_4_. J. Colloid Interface Sci..

[22] Pieterse J., Top H., Vollink F., Hoving K., van den Brink R. (2006). Selective catalytic reduction of NOx in real exhaust gas of gas engines using unburned gas: Catalyst deactivation and advances toward long-term stability. Chem. Eng. J..

[23] Li Z., Flytzani-Stephanopoulos M. (1999). Effects of water vapor and sulfur dioxide on the performance of Ce–Ag-ZSM-5 for the SCR of NO with CH_4_. Appl. Catal. B.

[24] Zhao J., Chen Y., Wang Y., Li Z., Nkinahamira F., Zhu R., Zhang J., Sun S., Zhu Y., Li H. (2023). The poisoning mechanism of H_2_O/SO_2_ to In/H-Beta for selective catalytic reduction of NO_x_ with methane. Appl. Catal. A.

[25] Zhang C., Xu G., Zhang Y., Chang C., Jiang M., Ruan L., Xiao M., Yan Z., Yu Y., He H. (2024). Designing a Ce/In-CHA OXZEO catalyst for high-efficient selective catalytic reduction of nitrogen oxide with methane. Appl. Catal. B.

[26] Kikuchi E., Ogura M., Terasaki I., Goto Y. (1996). Selective Reduction of Nitric Oxide with Methane on Gallium and Indium Containing H-ZSM-5 Catalysts: Formation of Active Sites by Solid-State Ion Exchange. J. Catal..

[27] Jing G., Li J., Yang D., Hao J. (2009). Promotional mechanism of tungstation on selective catalytic reduction of NO_x_ by methane over In/WO_3_/ZrO_2_. Appl. Catal. B.

[28] Chen K., Horstmeier S., Nguyen V.T., Wang B., Crossley S.P., Pham T., Gan Z., Hung I., White J.L. (2020). Structure and Catalytic Characterization of a Second Framework Al(IV) Site in Zeolite Catalysts Revealed by NMR at 35.2 T. J. Am. Chem. Soc..

[29] Chen K., Gan Z., Horstmeier S., White J.L. (2021). Distribution of Aluminum Species in Zeolite Catalysts: ^27^Al NMR of Framework, Partially-Coordinated Framework, and Non-Framework Moieties. J. Am. Chem. Soc..

[30] Fan B., Zhu D., Wang L., Xu S., Wei Y., Liu Z. (2022). Dynamic evolution of Al species in the hydrothermal dealumination process of CHA zeolites. Inorg. Chem. Front..

[31] Xing Y., Li G., Lin Z., Xu Z., Huang H., Zhu Y., Tsang S.C.E., Li M.M.J. (2023). *In situ* hierarchical pore engineering in small pore zeolite *via* methanol-mediated NH_4_F etching. J. Mater. Chem. A.

[32] Gao F., Washton N.M., Wang Y., Kollár M., Szanyi J., Peden C.H. (2015). Effects of Si/Al ratio on Cu/SSZ-13 NH_3_-SCR catalysts: Implications for the active Cu species and the roles of Brønsted acidity. J. Catal..

[33] Ma Y., Ding J., Yang L., Wu X., Gao Y., Ran R., Weng D. (2023). Flexible Al Coordination with H_2_O Explaining the Deviation of Strong Acid Amount from the Framework Al Content in Al-Rich SSZ-13. J. Phys. Chem. C.

[34] Schmidt C., Sowade T., Löffler E., Birkner A., Grünert W. (2002). Preparation and Structure of In-ZSM-5 Catalysts for the Selective Reduction of NO by Hydrocarbons. J. Phys. Chem. B.

[35] Gabrienko A.A., Arzumanov S.S., Moroz I.B., Prosvirin I.P., Toktarev A.V., Wang W., Stepanov A.G. (2014). Methane Activation on In-Modified ZSM-5: The State of Indium in the Zeolite and Pathways of Methane Transformation to Surface Species. J. Phys. Chem. C.

[36] Maunula T., Ahola J., Hamada H. (2006). Reaction mechanism and kinetics of NO_x_ reduction by methane on In/ZSM-5 under lean conditions. Appl. Catal. B.

[37] Wang D., Gao F., Peden C.H.F., Li J., Kamasamudram K., Epling W.S. (2014). Selective Catalytic Reduction of NO_x_ with NH_3_ over a Cu-SSZ-13 Catalyst Prepared by a Solid-State Ion-Exchange Method. ChemCatChem.

[38] Nasser G.A., Muraza O., Nishitoba T., Malaibari Z., Al-Shammari T.K., Yokoi T. (2019). OSDA-free chabazite (CHA) zeolite synthesized in the presence of fluoride for selective methanol-to-olefins. Microporous Mesoporous Mater..

[39] Meng X., Lian Z., Wang X., Shi L., Liu N. (2020). Effect of dealumination of HZSM-5 by acid treatment on catalytic properties in non-hydrocracking of diesel. Fuel.

[40] Resini C., Montanari T., Nappi L., Bagnasco G., Turco M., Busca G., Bregani F., Notaro M., Rocchini G. (2003). Selective catalytic reduction of NO_x_ by methane over Co-H-MFI and Co-H-FER zeolite catalysts: Characterisation and catalytic activity. J. Catal..

[41] Shen Q., Li L., He C., Zhang X., Hao Z., Xu Z. (2012). Cobalt zeolites: Preparation, characterization and catalytic properties for N_2_O decomposition. Asia-Pac. J. Chem. Eng..

[42] van Meerten S., Franssen W., Kentgens A. (2019). ssNake: A cross-platform open-source NMR data processing and fitting application. J. Magn. Reson..

