# Supplementary material for: Engineering In-Co3O4/H-SSZ-39(OA) Catalyst for CH4-SCR of NOx: Mild Oxalic Acid (OA) Leaching and Co3O4 Modification
Source: Molecules. 2024 Aug 7;29(16):3747. doi: 10.3390/molecules29163747 (PMC11357400; doi:10.3390/molecules29163747)
Supplement: Supplementary file 1 [file molecules-29-03747-s001.zip › molecules-3083805-supplementary.pdf]

Supporting Information for:

Engineering In-Co_3O_4/H-SSZ-39(OA) Catalyst for CH_4-SCR of NO_x: Mild Oxalic Acid (OA) Leaching and Co_3O_4 Modification

Guanyu Chen [1,†], Weixin Zhang [1,†], Rongshu Zhu [1,*], Yanpeng Chen [1], Minghu Zhao [1], Mei Hong [2,*]

1 State Key Laboratory of Urban Water Resource and Environment, Shenzhen Key Laboratory of Organic Pollution and Control, School of Civil and Environmental Engineering, Harbin Institute of Technology, Shenzhen, Shenzhen 518055, China

2 Guangdong Provincial Key Laboratory of Nano-Micro Materials Research, School of Advanced Materials, Peking University Shenzhen Graduate School (PKUSZ), Shenzhen 518055, China

[*] Correspondence: rszhu@hit.edu.cn (R.Z.); hongmei@pku.edu.cn (M.H.)

[†] These authors contributed equally to this work.

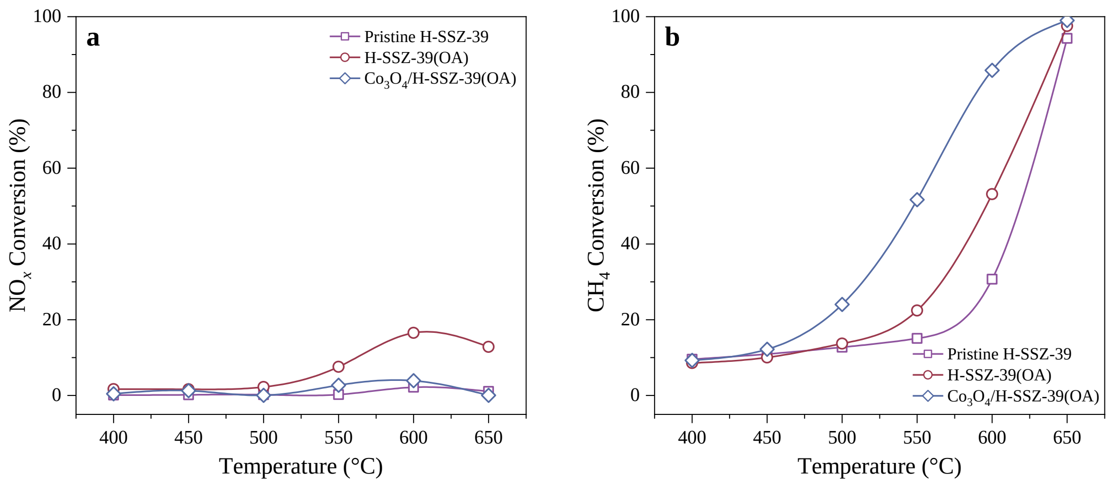

Figure S1. The CH_4-SCR deNO$_x$ performance of Pristine H-SSZ-39, H-SSZ-39(OA), and H-Co_3O_4-SSZ-39(OA) under dry conditions. Co_3O_4/H-SSZ-39(OA) with Co_3O_4: H-SSZ-39(OA) mass ratio of 1:30 was prepared in the same way as In-Co_3O_4/H-SSZ-39(OA) except that the indium nitrate aqueous solution was replaced with an equal volume of untrapure water. Reaction conditions: [NO] = 400 ppm, [CH_4] = 600 ppm, [O_2] = 10 vol%, Ar balance, GHSV = 24,000 h^{-1}.

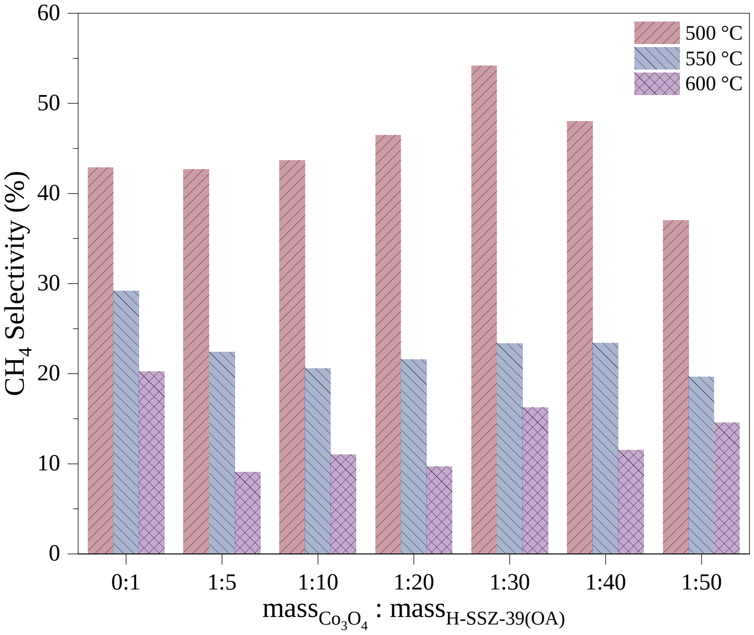

Figure S2. Effect of Co_3O_4 to H-SSZ-39(OA) mass ratio on CH_4 selectivity of the resulting catalysts under wet conditions. Reaction conditions: [NO] = 400 ppm, [CH_4] = 600 ppm, [O_2] = 10 vol%, [H_2O] = 5 vol%, Ar balance, GHSV = 24,000 h^{-1}.

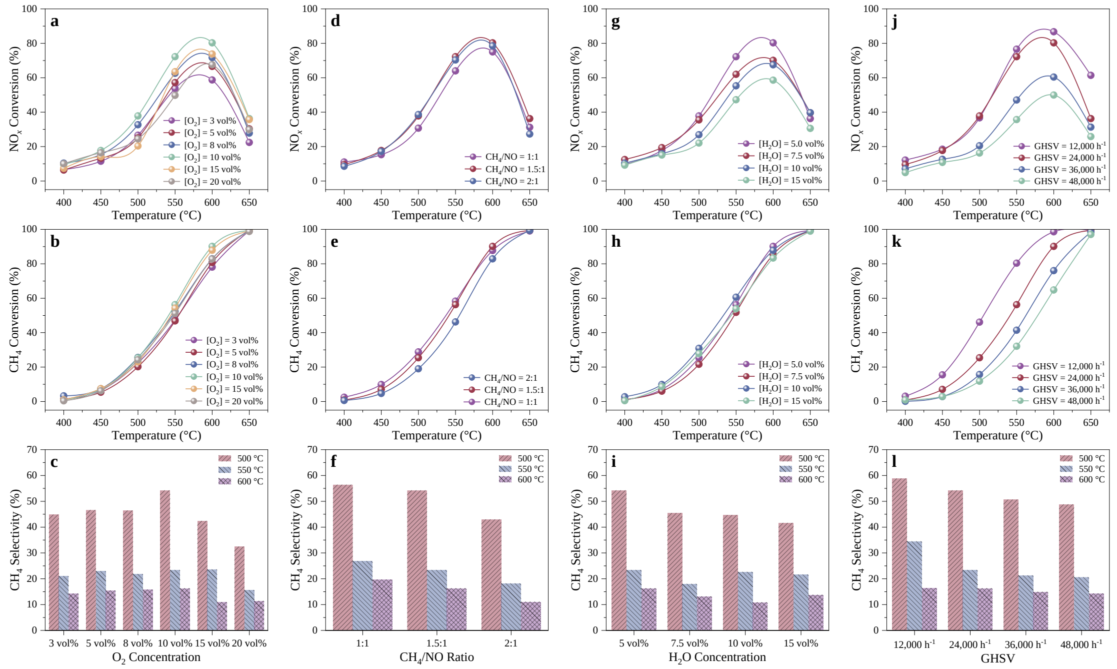

Figure S3. The CH_4-SCR deNO$_x$ performance of In-Co_3O_4/H-SSZ-39(OA) at different (**a–c**) O_2 concentrations, (**d–f**) CH_4/NO ratios, (**g–i**) water vapor concentrations, and (**j–l**) GHSVs. Reaction conditions in (**a–c**): [NO] = 400 ppm, [O_2] = 3–20 vol%, [CH_4] = 600 ppm, [H_2O] = 5 vol%, Ar balance, GHSV = 24,000 h^{-1}. Reaction conditions in (**d–f**): [NO] = 400 ppm, [O_2] = 10 vol%, [CH_4] = 400–800 ppm, [H_2O] = 5 vol%, Ar balance, GHSV = 24,000 h^{-1}. Reaction conditions in (**g–i**): [NO] = 400 ppm, [O_2] = 10 vol%, [CH_4] = 600 ppm, [H_2O] = 5–15 vol%, Ar balance, GHSV = 24,000 h^{-1}. Reaction conditions in (**j–l**): [NO] = 400 ppm, [O_2] = 10 vol%, [CH_4] = 600 ppm, [H_2O] = 5 vol%, Ar balance, GHSV = 12,000–48,000 h^{-1}.

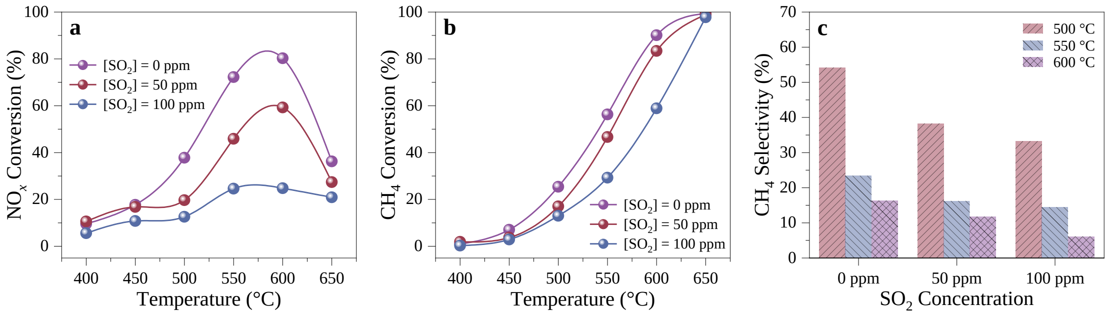

Figure S4. The CH_4-SCR deNO$_x$ performance of In-Co_3O_4/H-SSZ-39(OA) at different SO_2 concentrations and in the presence of 5 vol% H_2O. Reaction conditions: [NO] = 400 ppm, [CH_4] = 600 ppm, [O_2] = 10 vol%, [H_2O] = 5 vol%, [SO_2] = 0–100 ppm, Ar balance, GHSV = 24,000 h^{-1}.

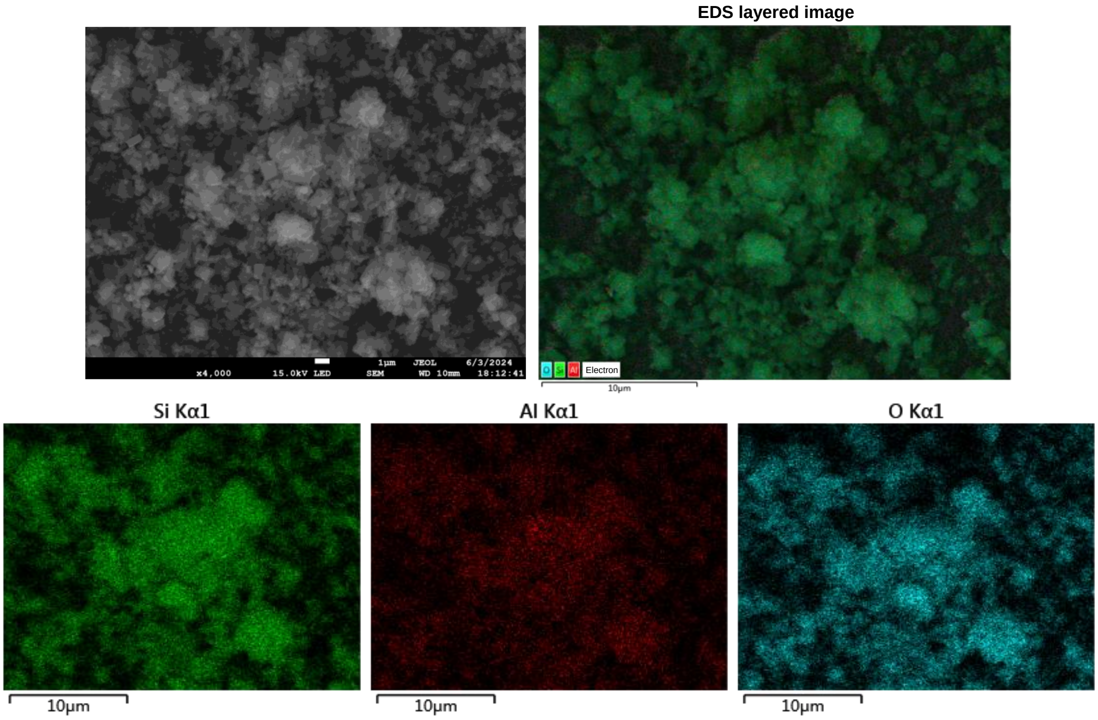

Figure S5. SEM-EDS mapping of Pristine H-SSZ-39.

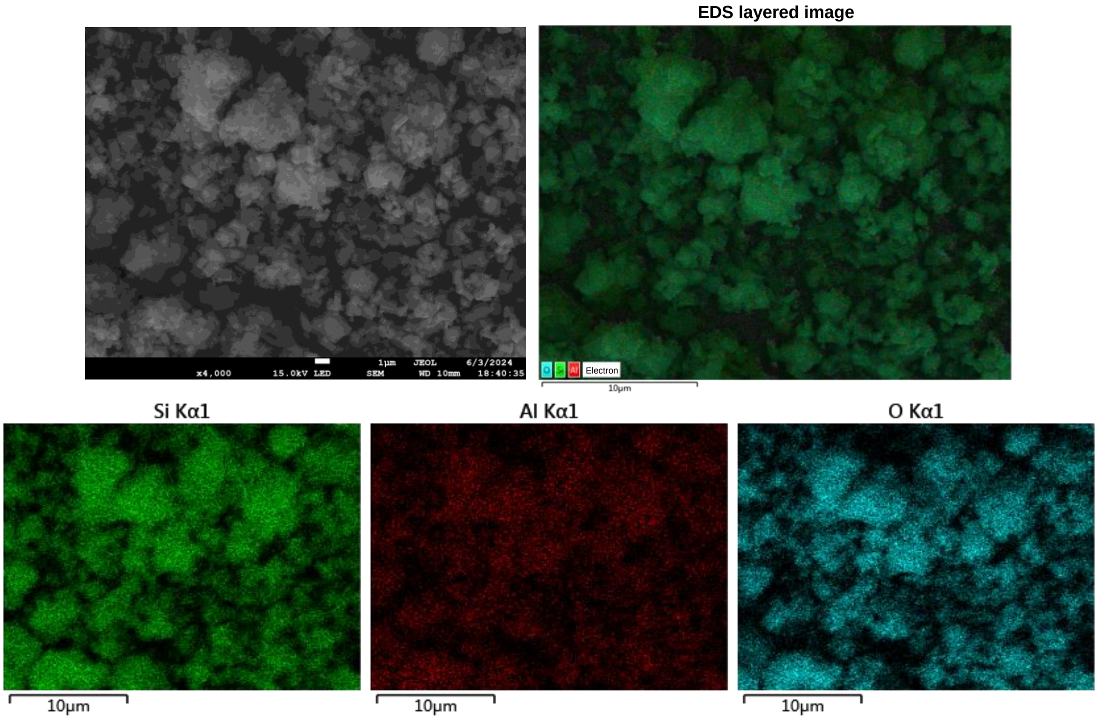

Figure S6. SEM-EDS mapping of H-SSZ-39(OA).

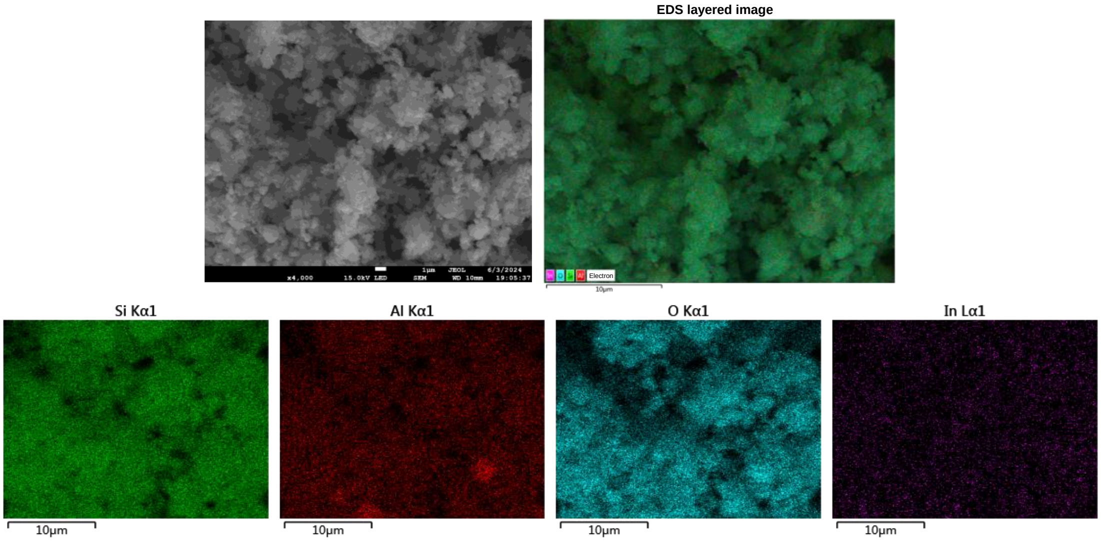

Figure S7. SEM-EDS mapping of In/H-SSZ-39(OA).

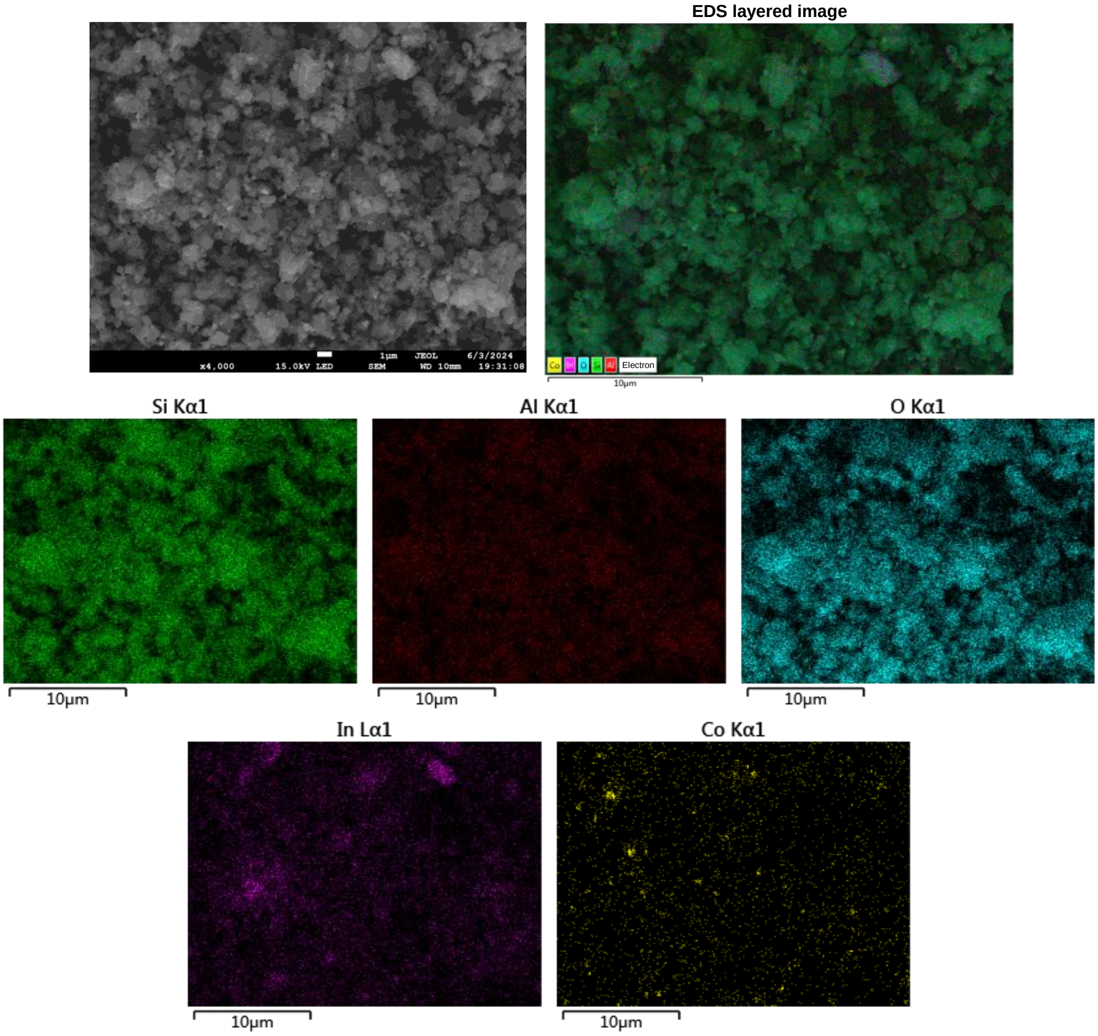

Figure S8. SEM-EDS mapping of In-Co_3O_4/H-SSZ-39(OA).

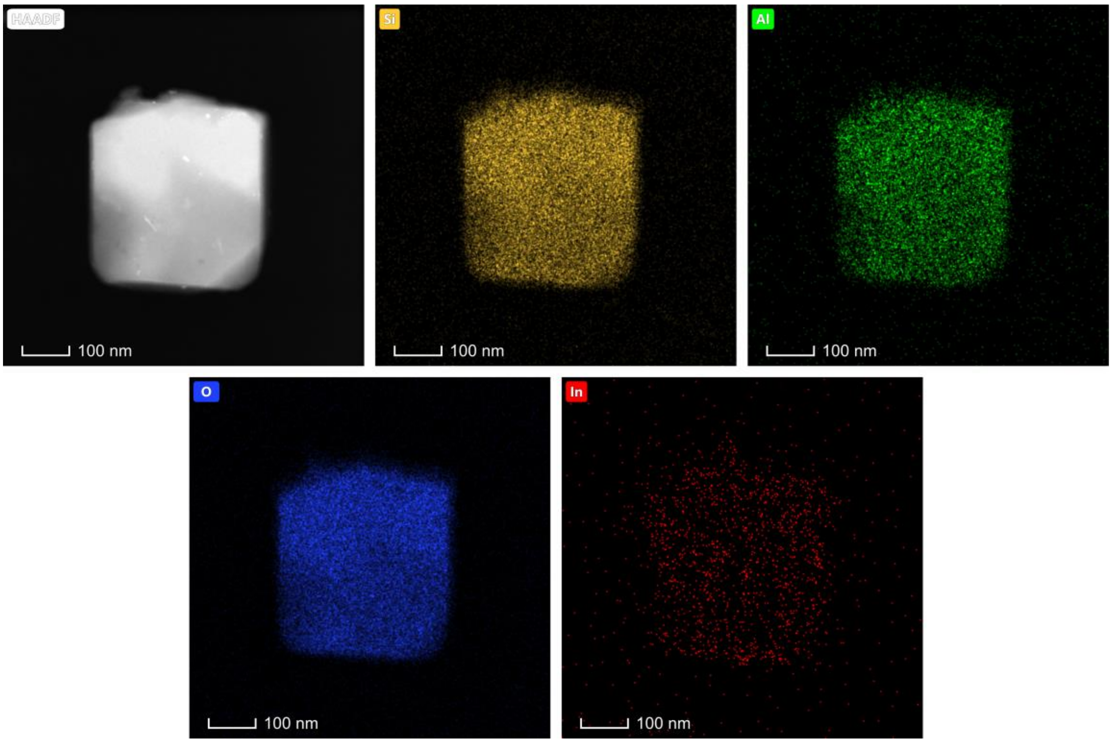

Figure S9. HAADF-STEM and elemental mapping images of In/H-SSZ-39(OA).

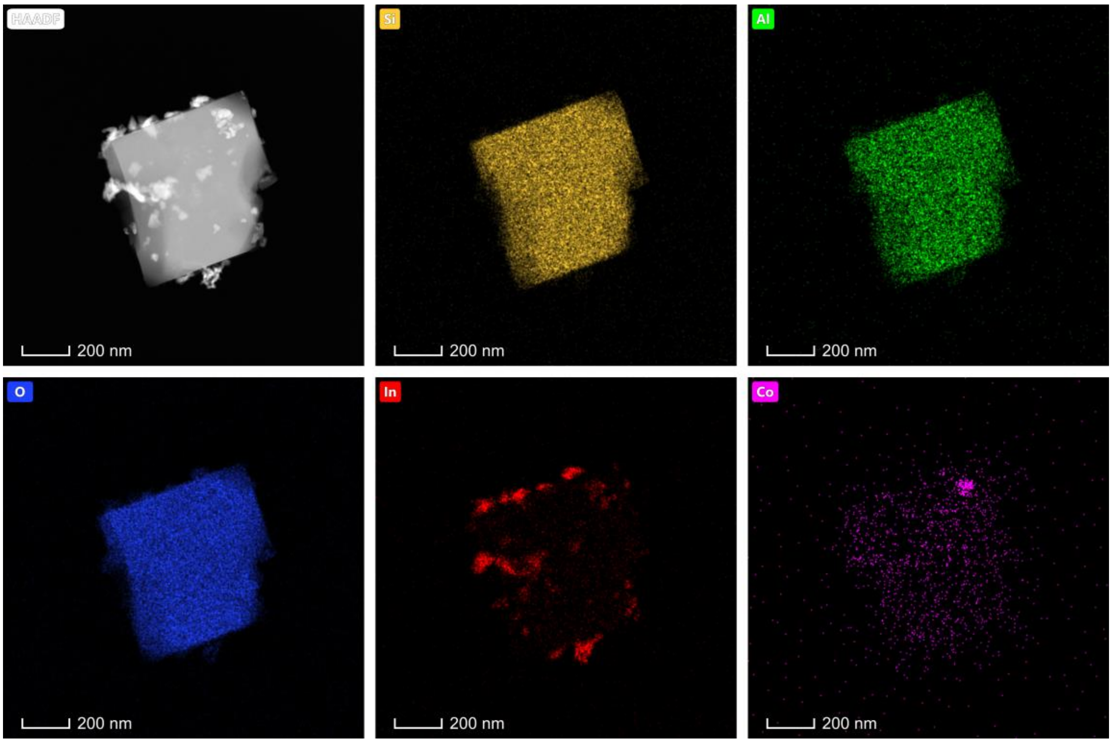

Figure S10. HAADF-STEM and elemental mapping images of In-Co_3O_4/H-SSZ-39(OA).

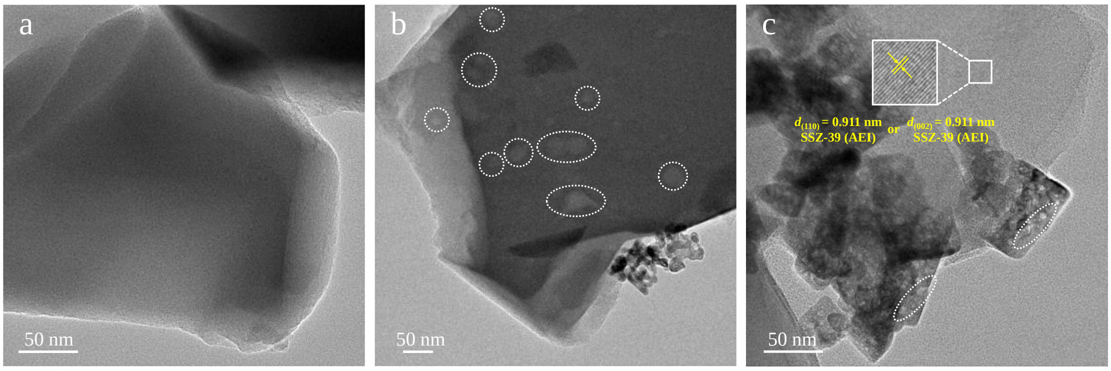

Figure S11. HRTEM images of (**a**) Pristine H-SSZ-39, (**b**) In/H-SSZ-39(OA), and (**c**) In-Co_3O_4/H-SSZ-39(OA).

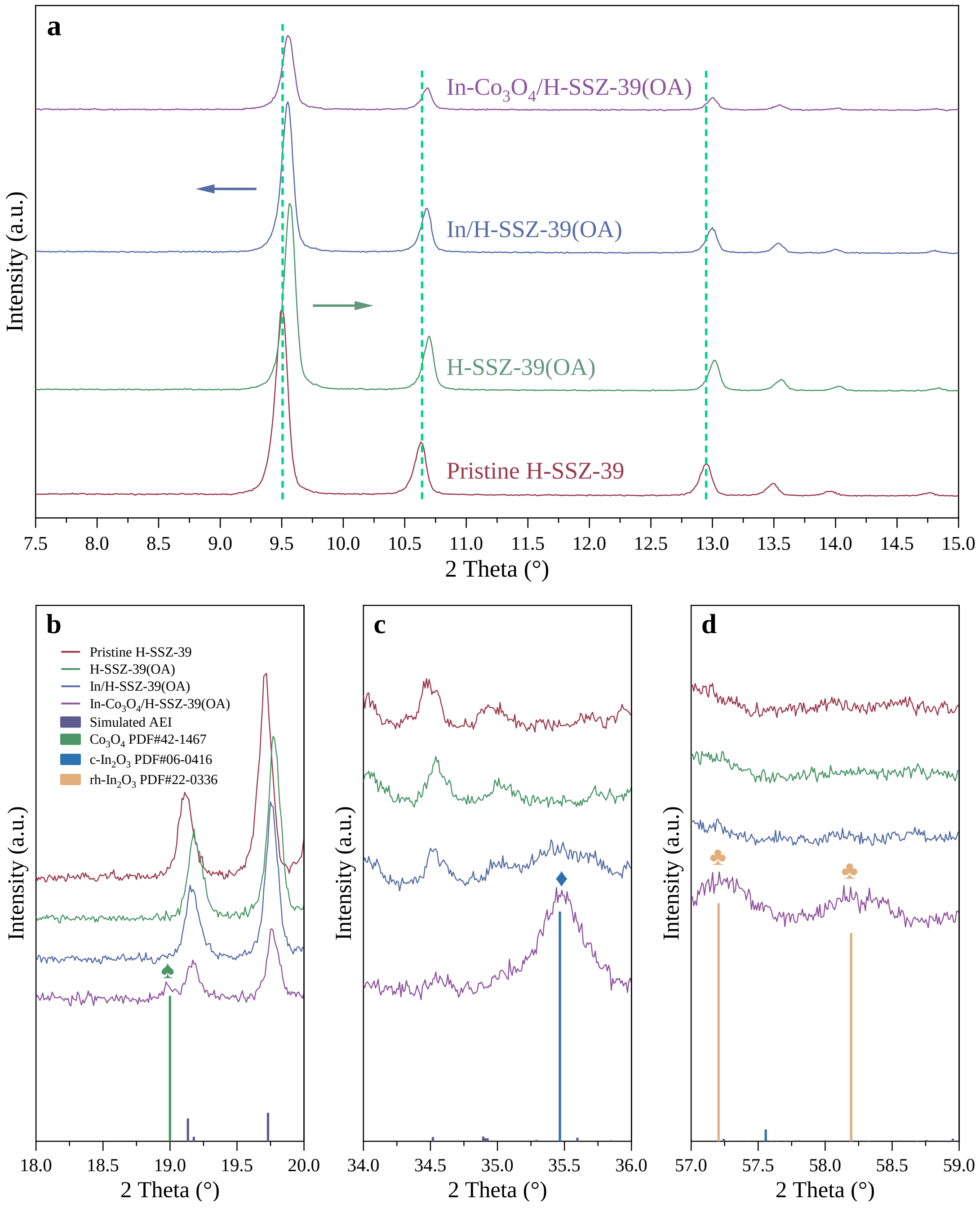

Figure S12. Locally enlarged PXRD patterns in the range of (**a**) 7.5–15.0°, (**b**) 18.0–20.0°, (**c**) 34.0–36.0°, and (**d**) 57.0–59.0° of Pristine H-SSZ-39, H-SSZ-39(OA), In/H-SSZ-39(OA), and In/H-Co_3O_4-SSZ-39(OA).

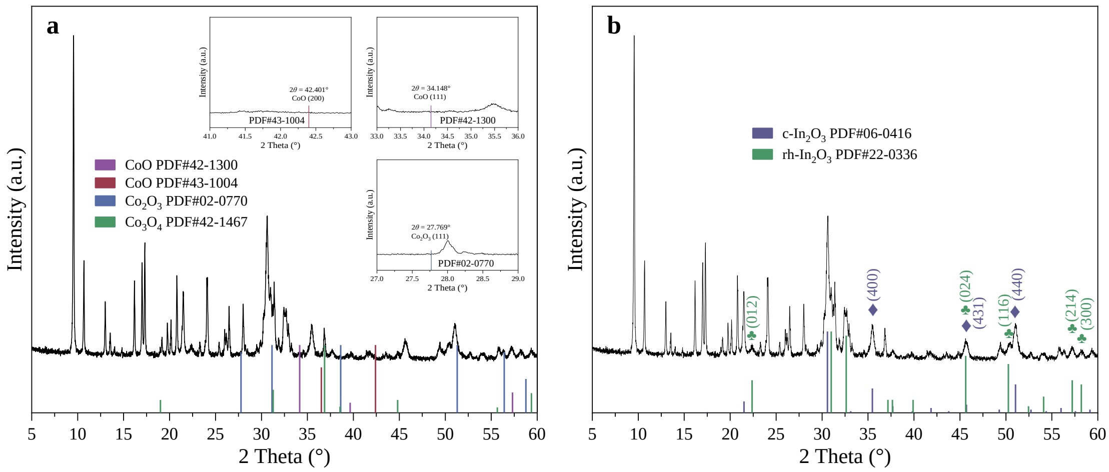

Figure S13. PXRD pattern of In-Co_3O_4/H-SSZ-39(OA) in comparison with standard PXRD patterns of (**a**) cobalt oxides and (**b**) indium oxides.

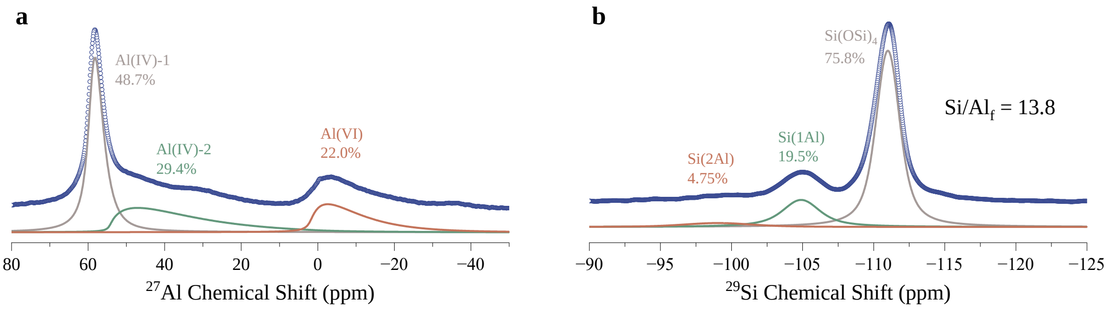

Figure S14. (**a**) ^{27}Al MAS SSNMR and (**b**) ^{29}Si MAS SSNMR spectra of H-SSZ-39(OA).

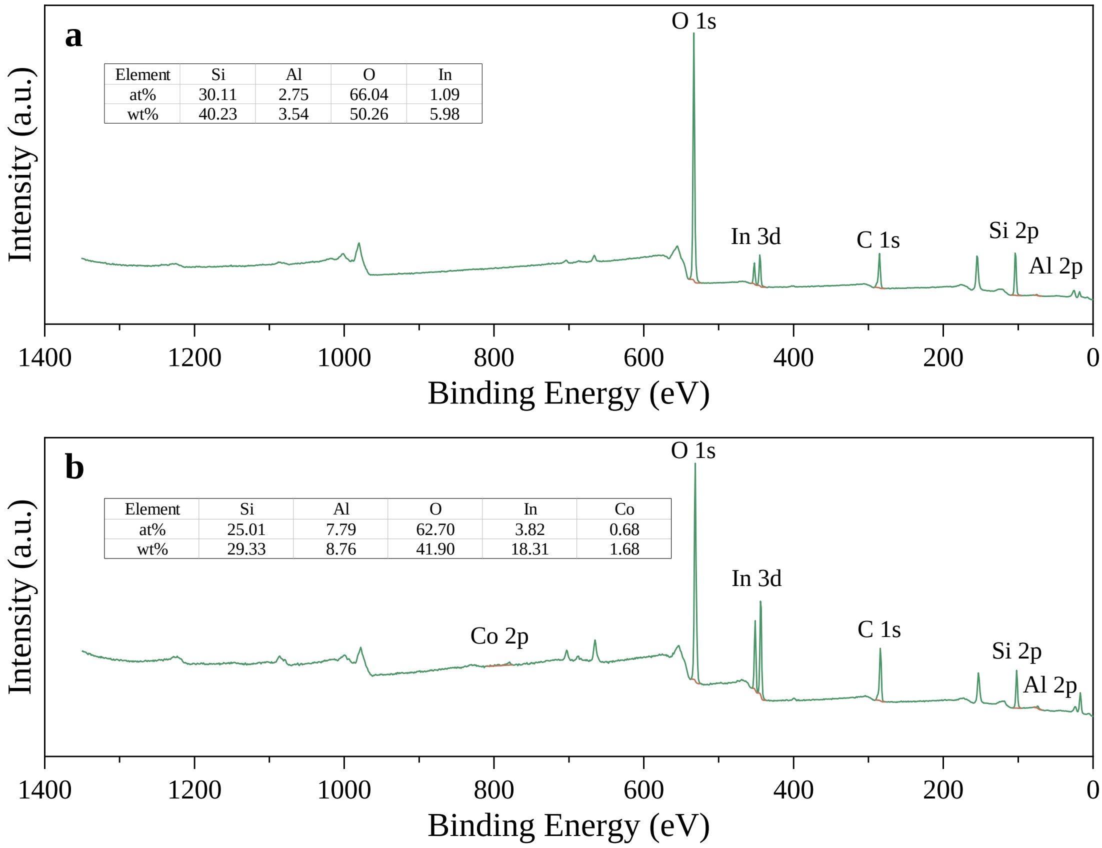

Figure S15. XPS survey spectra and corresponding surface element contents of (**a**) In/H-SSZ-39(OA) and (**b**) In-Co_3O_4/H-SSZ-39(OA).
